# AGE DIFFERENCES IN MILLING BEHAVIOR AND PERCEIVED STRESS DURING THE COVID-19 PANDEMIC

**DOI:** 10.1093/geroni/igae098.2249

**Published:** 2024-12-31

**Authors:** Reilly Burns, Jennifer Piazza, Pimbucha Rusmevichientong, Michele Wood

**Affiliations:** California State University Fullerton, Moreno Valley, California, United States; California State University Fullerton, Fullerton, California, United States; California State University Fullerton, Fullerton, California, United States; California State University Fullerton, Fullerton, California, United States

## Abstract

In a disaster, milling behavior refers to persistently seeking relevant information. It occurs most often when levels of emergency preparedness are low and the disaster is unexpected (Wood et al., 2012). Excessive milling can create a sense of vulnerability and lead to a decline in feelings of trust and readiness (Ejeta et al., 2015). One antidote to milling is effective communication and well-designed programs that increase levels of disaster preparedness. Unfortunately, such communication and programs are often ineffective for older adults (Brown & Haun, 2014). The current study examined age differences in knowledge and milling behavior during the COVID-19 pandemic. Participants included 425 individuals from Orange County, CA (18-87, mean age 47.02 years), who completed an online survey. Results from multiple regression analyses revealed that higher levels of milling were associated with higher levels of perceived stress, particularly among the older adults in the sample (B =.031, p =.026). Older adults also reported being less knowledgeable about what they can do to prepare for a pandemic (B = -.009, p =.044). Results remained significant after adjusting for education, race, and gender. Findings suggest that increasing older adults’ knowledge of a disaster through trusted sources may reduce milling behavior, which could decrease levels of stress in this population during a disaster.

